# Anthropometrics, cancer risks, and survival outcomes in adult patients with glioma – a systematic review and meta-analysis

**DOI:** 10.1007/s00701-025-06579-4

**Published:** 2025-07-10

**Authors:** Jaehyun Ahn, Joonseok Kim, Christopher Shin, Stephen Ahn

**Affiliations:** 1 College of Medicine, The Catholic University of Korea, Seoul, South Korea; 2 Department of Neurosurgery, College of Medicine, Seoul St. Mary’s Hospital, The Catholic University of Korea, 222 Banpodae-ro, Seocho-gu, Seoul, 06591 South Korea

**Keywords:** Glioma, Anthropometric measurements, Risk factors, Survival, Prognostic factor

## Abstract

**Purpose:**

The association between anthropometric measures including BMI, height and cancer has been widely discussed. However, the role of these in the development and prognosis of glioma remains controversial. We aimed to study these relationships.

**Methods:**

We followed the Preferred Reporting Items for Systematic Reviews and Meta-analyses (PRISMA) reporting guideline. Papers reporting relationship between anthropometric measures and the risk of glioma, both incidence and survival, were considered relevant. Those published until January 31, 2024, were selected from PubMed, EMBASE, and the Cochrane Library. Studies were evaluated according to the modified Newcastle Ottawa Scale. Hazard ratios, relative risks, and 95% confidence intervals were pooled and synthesized using a random effects model.

**Results:**

Among 940 screened articles, 23 were selected. Taller height was significantly associated with an increased risk of both glioma (HR per 10 cm, 1.19; CI, 1.16 to 1.23) and glioblastoma (HR per 10 cm, 1.25; CI, 1.18 to 1.31). Higher BMI was positively correlated with an increased risk of glioma, both in categorical (RR, 1.08; CI, 1.03 to 1.12) and continuous measures (HR per 5 kg/m^2^, 1.01; CI, 1.00 to 1.03). Glioblastoma demonstrated a higher incidence risk (HR per 5 kg/m^2^, 1.02; 95% CI 1.00 to 1.05) and better survival outcomes (HR 0.75; 95% CI 0.59 to 0.96) with increasing BMI.

**Conclusion:**

This study provides critical insights into the relationship between glioma and anthropometric measures. Glioma and glioblastoma were associated with these measures in terms of both incidence and survival. Further research is necessary to uncover the mechanisms and develop preventative or therapeutic strategies.

**Supplementary Information:**

The online version contains supplementary material available at 10.1007/s00701-025-06579-4.

## Introduction

Anthropometric measures, including height and body mass index (BMI), have been well-established to be associated with the development and prognosis of various diseases such as cardiovascular diseases [3], and have been studied particularly in relation to different types of cancers [48, 50]. Height was initially studied in breast cancer among women [4] and has since been explored in other cancers including kidney and colorectal cancer with consistent results [21]. In addition, obesity, often measured using BMI and sometimes waist circumference, has also become a well-researched risk factor in various type of cancer including colorectal, liver, and prostate cancer [28]. Interestingly, the concept of the"obesity paradox"has emerged, where obese cancer patients appear to have better survival outcomes [29].

However, in the case of glioma—the most common primary brain tumour and the most aggressive form of brain cancer, with a median overall survival of less than five years [58]—such studies are limited. Moreover, existing research presents conflicting results. For instance, five studies support the association between taller height and increased glioma risk [1, 7, 13, 35, 61, 62], while three do not [33, 34, 60]. Similarly, two studies demonstrate a significant relationship between BMI and glioma risk [2, 33], whereas eleven report no significant association [7, 8, 13, 26, 34, 37, 39, 49, 53, 60–62]. Additionally, four studies highlight the presence of the obesity paradox [9, 10, 43, 56], while one study refutes it [25].

In this context, we aim to elucidate the potential associations between body height and BMI with the risk and survival of patients with adult-type diffuse gliomas, including glioblastoma. This study comprehensively assesses existing evidence regarding the relationship between body height or BMI and the occurrence or survival of patients diagnosed with these tumours. By synthesizing available data, we firstly seek to provide insights into these controversial issues in glioma patients, which could inform future research into the pathobiological mechanisms underlying glioma and help develop novel preventive or therapeutic strategies.

## Methods

### Search strategy and selection criteria

We followed the Preferred Reporting Items for Systematic Reviews and Meta-analyses (PRISMA) reporting guideline [32]. A systematic search was conducted through three databases: PubMed, EMBASE, and the Cochrane Library. Articles published until December 31, 2024 were screened using the following search strategy: *(‘Glioma’ OR ‘Glioblastoma’) AND (‘Body Mass Index’ OR ‘Obesity’ OR ‘Body Weight’ OR ‘Overweight’ OR ‘Body Height’) AND (‘Survival’ OR ‘Mortality’ OR ‘Death’ OR ‘Risk’ OR ‘Risk Factors’ OR ‘Proportional Hazards Models’)*. Each of the MeSH terms above were connected with their synonyms using the Boolean operator *OR*. Only articles written in English were included.

Retrospective and observational studies (i.e., cohort and case–control) that reported the association of anthropometric measures with the risk of glioma occurrence or death were selected. Screening was not restricted by study setting, size, race, or country but was limited to adult patients with glioma or glioblastoma. Studies missing either necessary outcomes or full text were also excluded.

The study selection process was carried out in two stages, ensuring a rigorous approach. First, titles and abstracts were carefully screened; then, selected full-text articles were included based on the pre-defined selection criteria. This screening process was conducted by three independent authors (CS, JA, JK). Every article was evaluated by two of these authors (CS and JA, JA and JK, or JK and CS). Disagreements were resolved through discussion among three authors, including a third reviewer who did not perform the evaluation. The reference lists of identified articles were manually searched.

### Data collection and quality assessment

Data were collected independently by using a predesigned spreadsheet. Collected items included authors, year of publication, study type, subject population, mean age, number of subjects, region, tumour type (glioma, glioblastoma, or both), timeframe for follow-up, cutoff of anthropometric measures, and outcomes (i.e., hazards ratio, relative risk, overall survival).

The quality of every article was assessed based on modified Newcastle Ottawa Scale (NOS; range 1–9, with 1–3 indicating low quality, 4–6 indicating moderate quality, and 7–9 indicating high quality) [59]. In both case–control and cohort studies, age was identified as the most important factor for comparability. A follow-up period of 5 years and a follow-up rate of 80% were deemed adequate. As the risk and survival of paediatric glioma patients are beyond the interest of this study, cohorts consisting of average adults were considered representative. Each study underwent assessment by two independent researchers (CS and JA, JA and JK, or JK and CS). Disagreements were resolved through discussion among three researchers, including a third individual who did not perform the assessment.

### Statistical analysis

For the analysis of the relationship between categorical BMI and glioma risk, we sought to maximise inclusivity by employing relative risk (RR) as a measure of association. For investigations into other relationships (continuous BMI and glioma risk, height and glioma risk, BMI and glioma survival) we exclusively considered studies reporting fully adjusted hazard ratios (HR). To standardise the discrepancy in set endpoints, we adopted the inverse value of reported hazard ratios from several studies. Pooled HRs, RRs, and their 95% confidence intervals (CIs) were determined using random-effects meta-analysis approach with generic inverse-variance method to integrate effect sizes from heterogeneous studies. For height, the effect of continuous increase of 10 cm was analysed. The dichotomous difference at cutoff of 25 kg/m^2^ and continuous increase of 5 kg/m^2^ was analysed for BMI and tumour risk. Meanwhile, the relationship between survival and BMI was obtained at dichotomous comparison between high BMI versus low BMI due to discrepancies of BMI cutoff across studies. The degree of heterogeneity across studies was evaluated using the I^2^ statistic. A common-effects model was applied when heterogeneity was low (I^2^ ≤ 50%), while a random-effects model was used in cases of substantial heterogeneity (I^2^ > 50%). To explore potential sources of heterogeneity, we conducted predefined subgroup analyses based on disease type (all-grade glioma, high-grade glioma, glioblastoma) and BMI cutoff. Subgroup differences were evaluated using the χ^2^ test. Funnel plots were visually inspected to assess the risk of publication bias. All statistical analyses were two-sided with significance level set at *p*-value < 0.05 and were performed using ‘R’ software version 4.0.3 (R Foundation for Statistical Computing, 2020).

## Results

Initial database search yielded 1049 articles, 34 (3.2%) of which met the inclusion criteria for detailed full-text review. Among them, eleven studies were excluded for one of the following reasons: unavailable outcome, unavailable number of subgroup patients, inappropriate timing of anthropometric measurement, and outdated definition of brain tumour (Supplementary Table S1). Additional 18 studies were identified through citation searching; however, none met the inclusion criteria. As a result, 23 studies were included in our meta-analysis. (Supplementary Fig. S1) Descriptive data for these studies are listed in Table 1. The mean NOS score was 7.13 (median, 7; range 5–9), indicating that the overall quality of the articles was high (Table 2).

**Table 1:** Characteristics of included studies

Authors	Year	Study type	Subject	Age (y, mean)	Population (N)	Region	Tumor type	Timeframe for follow-up
Moseeva et al. [37]	2024	Retrospective cohort	Mayak Production Association workers, exposed to radiation	NA (NA)	22,377	Russia	Glioma	Start: 1964–1999 End: December 2018
Sang et al. [49]	2023	Retrospective cohort	Adult diabetes patients enrolled in NHIS database	NA (57.5)	1,893,057	South Korea	Glioma	Start: January 2009 End: December 2018
Shao et al. [53]	2022	Prospective cohort	PLCO participants	42–78 (NA)	140,270	United States	Glioma	Start: 1993–2001 End: December 2009 Median follow-up: 12.04 years
Ahn et al. [1, 2]	2021	Retrospective cohort	Adult patients enrolled in NHIS database	20–80 (51.3)	6,833,744	South Korea	Glioma	Start: January 2009 End: December 2017 Median follow-up: 7.3 years
Cha et al. [10]	2021	Retrospective cohort	Primary diagnosis of GBM at Seoul St. Mary’s Hospital	20–85 (61.0)	177	South Korea	GBM	Start: August 2008 End: December 2018 Mean follow-up: 19.2 months
Valente Aguiar et al. [56]	2021	Retrospective cohort	Primary diagnosis of GBM at CHUSJ	NA (60 ^a^)	193	Portugal	GBM	Start: 2011 End: 2017 Median follow-up: 17.3 months
Ogawa et al. [39]	2020	Prospective cohort	JPHC participants	40–69 (51.8)	102,925	Japan	Glioma	Start: 1990, 1993 End: December 2012 Median follow-up: 18.1 years
Bertoli et al. [8]	2018	Cross-sectional	Case: primary diagnosis of HGG at clinical neuro-oncology unit of FINCB Control: propensity-matched random selection from ICANS database	NA (50)	Case: 51 Control: 51	Italy	HGG	Enrollment: March 2015-December 2015
Cote et al. [13]	2018	Prospective cohort	Female nurse, Male health professionals	30–75 (46.4)	173,096	United States	Glioma, GBM	Start: 1976, 1986 End: June 2014, February 2015 Median follow-up: 34.2, 23.6 years
Kabat et al. [26]	2018	Prospective cohort	Post-menopausal women	50–79 (NA)	161,119	United States	Glioma, GBM	Start: 1993–1998 Median follow-up: 17.8 years
Potharaju et al. [43]	2018	Retrospective cohort	Primary diagnosis of GBM	18–82 (56.0 ^a^)	392^b^	India	GBM	Start: January 2008 End: June 2016 Median follow-up: 48.6 months
Cata et al. [9]	2017	Retrospective cohort	Primary diagnosis of GBM at M.D. Anderson Cancer Center	NA (56.63)	381	United States	GBM	Start: January 2006 End: July 2015
He et al. [24]	2017	Retrospective cohort	Primary diagnosis of HGG at SYSUCC	5–78 (45.0 ^a^)	331	China	HGG	Start: January 2001-July2014 End: October 2015
Wiedmann et al. [61, 62]	2017	Prospective cohort	Tuberculosis screening campaign participants	14–80 (43.4)	1,855,333	Norway	Glioma, GBM	Start: 1963–1975 End: December 2011
Little et al. [33]	2013	Case–control	Primary diagnosis of glioma within 3 months	25–92 (55.5)	Case: 1,111 Control: 1,096	United States	Glioma	Enrollment: December 2004-June 2012
Siegel et al. [54]	2013	Retrospective cohort	Primary diagnosis of HGG within 3 months	25–92 (57)	853	United States	HGG	Start: February 2005-March 2012
Wiedmann et al. [60]	2013	Retrospective cohort	HUNT participants	20–101 (47.5)	74,242	Norway	Glioma	Start: 1984–1986 End: December 2008 Median follow-up: 23.5 years
Michaud et al. [34]	2011	Prospective cohort	EPIC participants	35–70 (52.2)	380,775	Europe	Glioma	Start: 1991–2000 Mean follow-up: 8.4 years
Jones et al. [25]	2010	Prospective cohort	Primary diagnosis of GBM at UCSF, DUMC	NA (58)	1,259^c^	United States	GBM	Start: January 1991–2008 Median follow-up: 40 months
Moore et al. [35]	2009	Prospective cohort	AARP members	50–71 (62)	499,437	United States	Glioma	Start: 1995–1996 End: December 2003
Benson et al. [7]	2008	Prospective cohort	Middle-aged women	50–65 (55.9)	1,249,670	United Kingdom	Glioma	Start: May 1996-March 2001 End: December 2005

*a* median age.

*b* 249 out of 392 patients were included in the analysis.

*c* 60% of cases were included in the analysis.

*AARP *American Association of Retired Persons; *CHUSJ*, Centro Hospitalar Universitário São João; *EPIC*, European Prospective Investigation into Cancer and Nutrition; *FINCB*, Foundation of the Carlo Besta Neurological Institute Milan; *GBM*, glioblastoma multiforme; *HGG*, high grade glioma; *HUNT*, The Nord–Trøndelag Health Study; *ICANS*, International Center for the Assessment of Nutrtional Status; *JPHC*, The Japan Public Health Center-Based Prospective Study; *NHIS*, Korean National Health Insurance System; *PLCO*, The Prostate, Lung, Colorectal, and Ovarian Cancer Screening Trial; *SYSUCC*, Sun Yatsen University Cancer Center;

**Table 2:** Quality assessment for included studies

Authors	Year	Selection				Comparability	Exposure/Outcome^a^			Quality score^c^
		1	2	3	4		1	2	3	
Moseeva et al. [37]	2024	☆	★	★	☆	★★	★	★	★	7
Sang et al. [49]	2023	☆	★	★	★	★★	★	★	★	8
Shao et al. [53]	2022	★	★	☆	★	★★	☆	★	★	7
Ahn et al. [2]	2021	★	★	☆	★	★★	★	★	★	8
Ahn et al. [1]	2021	★	★	★	★	★★	★	★	★	9
Cha et al. [10]	2021	★	★	☆	★	★★	★	★	★	8
Valente Aguiar et al. [56]	2021	★	★	★	☆	☆☆	★	★	★	6
Ogawa et al. [39]	2020	★	★	☆	☆	★★	★	★	★	7
Bertoli et al. [8]	2018	★	★	☆	★	★★	★	★	☆	7
Cote et al. [13]	2018	☆	★	☆	☆	★☆	★	★	★	5
Kabat et al. [26]	2018	☆	★	★	★	★★	☆	★	☆	6
Potharaju et al. [43]	2018	★	★	★	☆	★★	★	☆	★	7
Cata et al. [9]	2017	★	★	★	☆	★★	★	☆	★	7
He et al. [24]	2017	★	★	★	☆	★★	★	☆	☆	6
Wiedmann et al. [61, 62]	2017	★	★	★	★	★★	★	★	★	9
Little et al. [33]	2013	★	★	★	★	★★	★	★	☆	8
Siegel et al. [54]	2013	★	★	★	★	★★	☆	★	☆	7
Wiedmann et al. [60]	2013	★	★	★	★	★★	★	★	★	9
Michaud et al. [34]	2011	★	★	☆	★	★★	★	★	★	8
Jones et al. [25]	2010	★	★	☆	☆	★★	★	☆	★	6
Moore et al. [35]	2009	☆	★	☆	★	★★	★	★	☆	6
Benson et al. [7]	2008	☆	★	☆	★	★★	★	★	★	7

a assessed exposure for case-control studies and outcomes for cohort studies

b applicable to cohort studies

c assessed according to modified Newcastle Ottawa scale (range 1–9, a score of 1–3 indicates low quality, 4–6 indicates moderate quality, and 7–9 indicates high quality)

NA, not applicable;

### Height and risk of glioma, glioblastoma

**Fig. 1:**
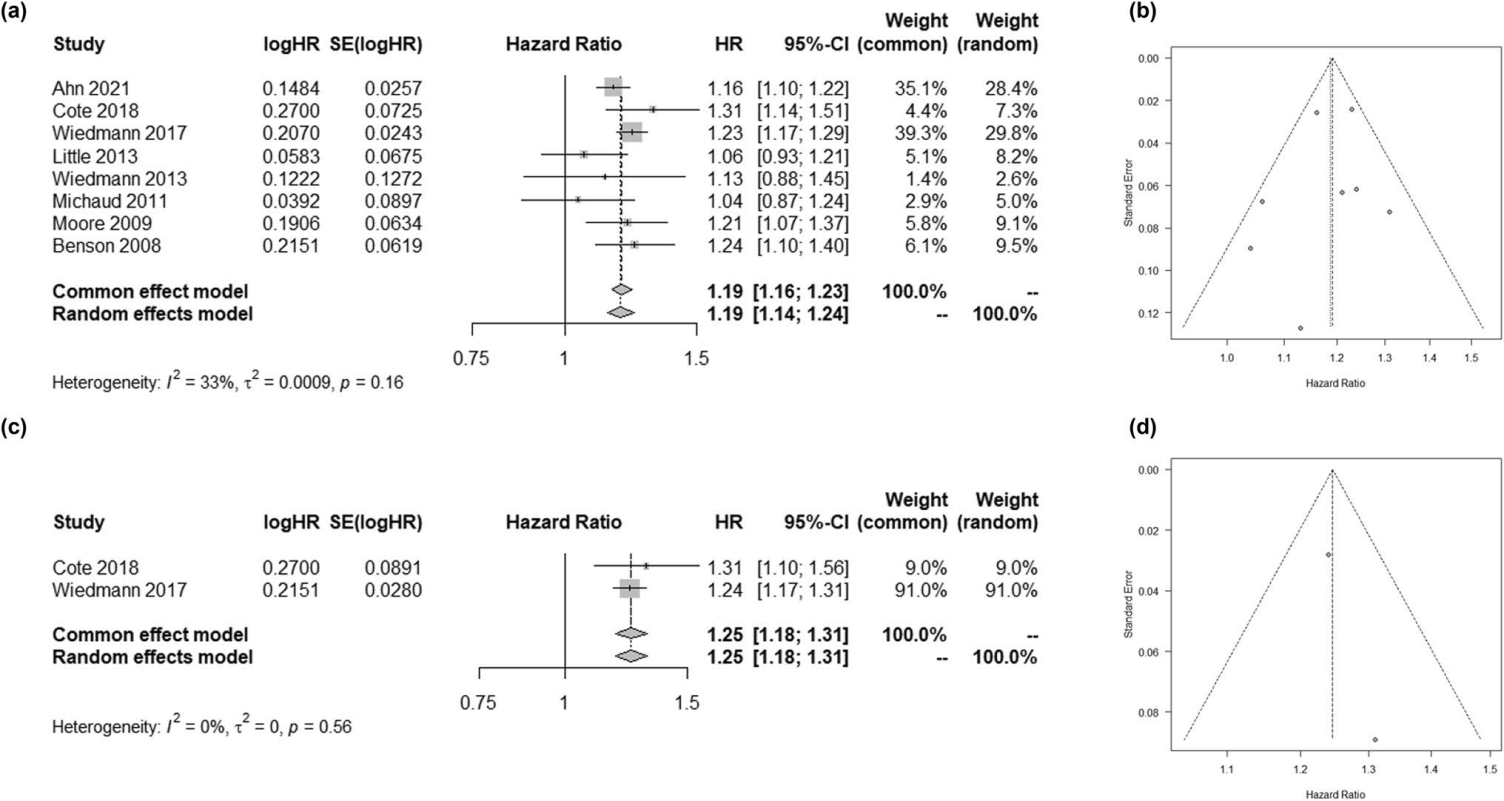
Shown are the association between height and the risk of (**a**) glioma occurrence or (**c**) glioblastoma occurrence. Panel (**b**) and (**d**) are corresponding funnel plots

A total of eight studies reported data on height and glioma occurrence (Supplementary Table S2) [1, 7, 13, 33–35, 60–62], with two of these studies specifically addressing the risk of glioblastoma [13, 61]. All studies indicated a positive association between height and the risk of both glioma (HR per 10 cm, 1.19; 95% CI, 1.16–1.23; Fig. 1a, b) and glioblastoma (HR per 10 cm, 1.25; 95% CI, 1.18–1.31; Fig. 1c, d). To clarify, the results from two papers were combined, as each analysis was conducted by the same research group using the same cohort [61, 62].

### BMI and risk of glioma, glioblastoma

**Fig. 2:**
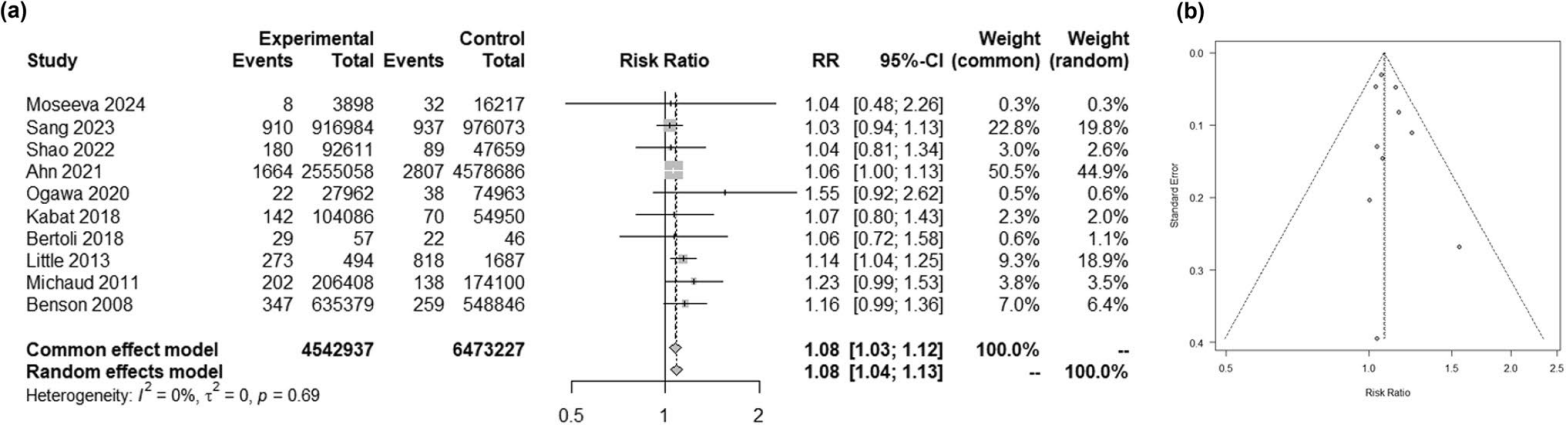
The association between BMI as a categorical variable and the risk of glioma occurrence. (**a**) Forest plot showing the association. (**b**) Funnel plot

**Fig. 3:**
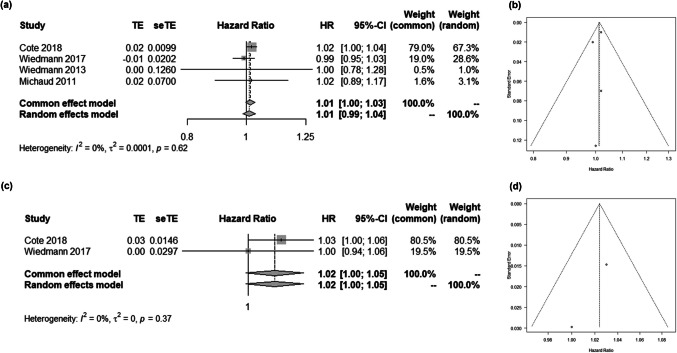
Shown are the association between BMI as a continuous variable and the risk of (**a**) glioma occurrence or (**c**) glioblastoma occurrence. Panel (**b**) and (**d**) are corresponding funnel plots

We included a total of 13 studies examining the relationship between BMI and glioma risk (Supplementary Table S3) [2, 7, 8, 13, 26, 33, 34, 37, 39, 49, 53, 60–62]. These studies investigated either the comparison of glioma risks across categorical BMI levels or the impact of every 5 kg/m^2^ increase in continuous BMI. Given the variability in BMI level cutoffs across studies, we opted to measure RRs for the analysis of categorical BMI and glioma risk. While previous research has yielded conflicting findings regarding the RR of BMI ≥ 25 kg/m^2^ compared to BMI < 25 kg/m^2^, our pooled RR analysis demonstrated statistical significance (RR 1.08; 95% CI 1.03–1.12; Fig. 2a, b). Similarly, consistent results emerged in HRs for both glioma (HR 1.01; 95% CI 1.00–1.03; Fig. 3a, b) and glioblastoma (HR 1.02; 95% CI 1.00–1.05; Fig. 3c, d) risk with every 5 kg/m^2^ increase in continuous BMI.

### BMI and glioblastoma survival

Among studies that investigated the impact of BMI on survival in glioblastoma or glioma patients, seven reported hazard ratios [9, 10, 24, 25, 43, 54, 56]. Two studies were excluded due to the absence of reported hazard ratios (Supplementary Table S4) [52, 57]. The analysis included five studies focused on glioblastoma patients [9, 10, 25, 43, 56], with an additional two studies involving high-grade glioma patients included in sensitivity analysis [24, 54]. Detailed information and results from each study are summarized in Supplementary Table S5.

**Fig. 4:**
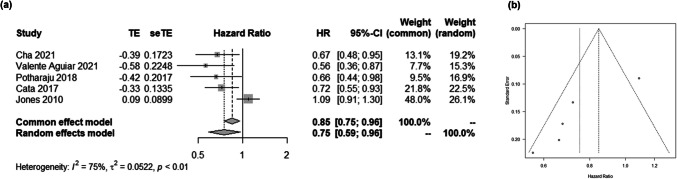
The association between BMI and glioblastoma survival. (**a**) Forest plot showing the association. (**b**) Funnel plot

The pooled data revealed an association between higher BMI and improved survival outcomes in patients with glioblastoma (HR 0.75; 95% CI 0.59–0.96; Fig. 4a). The Funnel plot showed asymmetry (Fig. 4b). Sensitivity analysis indicated a nonsignificant relationship between higher BMI and survival outcomes in high grade glioma patients (HR 0.84; 95% CI 0.67–1.07; Supplementary Fig. S2).

## Discussions

Before our study, the relationship between height or BMI and glioma risk had been suggested, but the results were inconsistent, as shown in Supplementary Tables S2 and S3. To summarise, five studies have supported the association between height and glioma with HRs ranging from 1.16 to 1.31 [1, 7, 13, 35, 61, 62], while three did not [33, 34, 60]. Similarly, for BMI and glioma risk, two studies showed support with HRs ranging from 1.14 to 1.20 [2, 33], while eleven found no significant association [7, 8, 13, 26, 34, 37, 39, 49, 53, 60–62]. A previous meta-analysis which includes fewer studies also found insignificant relationship [38]. Through our meta-analysis, we demonstrated a combined, statistically significant relationship. Specifically, for height, the pooled HR was 1.19 (95% CI: 1.16–1.23) for glioma risk, while for BMI, the HR was 1.08 (95% CI: 1.03–1.12), indicating a positive association of both height and BMI with glioma occurrence. Similar relationship was observed when analysing studies for glioma and glioblastoma risk per unit increase in BMI, with HRs of 1.01 (95% CI: 1.00–1.03) for glioma and 1.02 (95% CI: 1.00–1.05) for glioblastoma [13, 33, 34, 60–62], thereby underscoring the importance of BMI as a modifiable risk factor for these tumours. To our knowledge, this is the first meta-analysis to synthesise these findings comprehensively. These results are further supported by consistent associations observed in various types of cancer [4, 5, 11, 21, 22, 44].

This meta-analysis also demonstrated the association between higher BMI and improved survival outcomes in glioblastoma patients by synthesizing previously conflicting studies whose reported HR range from 0.56 to 1.09 [9, 10, 25, 43, 56]. This phenomenon, often referred to as the “obesity paradox” because it contradicts the relationship observed in the general population [16], has been identified in only a limited number of cancers [31, 41]. Nonetheless, in this analysis, the obesity paradox was observed in glioblastoma patients, with an HR of 0.75 (95% CI: 0.59–0.96). The sensitivity analysis indicated a nonsignificant difference in survival between higher and lower BMI groups in combined high-grade glioma patients, in contrast to findings in glioblastoma, suggesting that the impact of BMI on survival may vary according to tumour grade.

### Pathobiological mechanism

While biological mechanism behind each relationship is not yet fully understood, there are some studies that support our results. One possible explanation for the association between taller height and increased risk of glioma and glioblastoma is that taller individuals may have a greater number of cells, thereby increasing the likelihood of malignant transformation. This hypothesis is supported by studies showing a positive association between higher intracranial volume and glioma risk [20]. Research on insulin-like growth factor (IGF) and related proteins (i.e., IGF receptors and IGF-binding proteins (IGFBPs)) provides another plausible explanation and offers hope for a novel target in cancer therapy [12, 36, 40, 45]. A number of in vitro and in vivo experiments supporting this hypothesis in glioblastoma have been published. High levels of IGF-related proteins have been found in glioblastoma [17, 51], and tumour growth was inhibited when these proteins were targeted [14, 46, 47, 55, 63, 64]. More recently, efforts are being made to exploit the IGF system by particularly targeting IGFBP-2, which is gaining attention due to its presence in cancer cells and absence in normal mature brain cells [6, 18, 30]. In our meta-analysis, we found that GBM was slightly more associated with tall stature than glioma, providing further support for the potential role of IGF-related proteins in gliomagenesis. However, the IGF hypothesis raises questions—particularly regarding the time lag between peak IGF secretion earlier in life and the peak incidence of glioma later in life—which warrants further investigation.

One of proposed mechanisms for BMI and glioma risk is pro-inflammatory state associated with increased body weight, which may contribute to tumorigenesis. Elevated body mass and hyperglycaemia activate pro-inflammatory pathways via the receptor for advanced glycation end products (RAGE), potentially enhancing glioma growth by upregulating RAGE expression and suppressing antitumor immune responses [65]. Another is genetic alterations related to higher BMI can lead to an increased development of malignancies. The fat mass and obesity-associated gene (FTO) is considered to be one of the key genetic contributors [15, 27].

For the obesity paradox observed in this study, various methodological and biological mechanisms have been proposed, including less aggressive tumour biology, better treatment response, and increased energy reserves [29]. This biological perspective is supported by the association of sarcopenia with poor survival outcomes which has been observed in glioblastoma patients [19]. Another possible explanation is that underweight patients, who tend to experience significantly worse outcomes compared to normal-weight individuals [54], may have skewed the analysis. Their inclusion in the lower BMI group could have exaggerated or falsely depicted the relationship between obesity and improved survival outcomes, potentially due to lower muscle mass [23].

### Limitation

Our study has several limitations that warrant cautious interpretation. The retrospective design of this study leaves certain confounders, such as socio-economic status and comorbidities, unaddressed [42]. However, most of the HRs and RRs included in this analysis were adjusted for key variables such as age and sex, which partially mitigate this concern. Another limitation is that only two studies are included in the analysis of height and glioblastoma risk [13, 61, 62], which is too small to draw a definitive conclusion. This limitation underscores the need for further research to confirm the relationship between height and glioblastoma. For the analysis of BMI and the risk of glioma occurrence, the BMI data in each study were not obtained at the same timeframe. Additionally, abdominal obesity, which interestingly has been shown to have a stronger association with glioma development, was beyond the scope of this analysis [2]. Significant heterogeneity among studies on BMI and survival is a concern. This may partly stem from variations in BMI group definitions and whether underweight individuals were included in the lower BMI category [9, 10, 25, 43, 56]. Differences in tumour biology also contribute to this heterogeneity, as shown in a previous study [57]. Another limitation is the potential for reverse causation, as glioma may have influenced patients’ BMI. Additionally, the presence of possible publication bias, as suggested by the Funnel plot, highlights the need for cautious interpretation of these result. It is also noteworthy that two studies that were excluded as summarised in Supplementary Table S5, reported conflicting findings [52, 57]. Therefore, further research is essential to better understand the true effect of BMI on survival outcomes.

## Conclusion

Our meta-analysis offers valuable insights into the nuanced relationship between obesity and the risk or survivorship of glioma and glioblastoma. The findings confirm that taller height is associated with an increased risk of both glioma and glioblastoma, while higher BMI correlates with an elevated risk of glioma. Additionally, we observed a link between higher BMI and improved survival outcomes. However, larger, population-based studies are required to fully validate these associations. Additional research is also warranted to reveal precise biological mechanisms.

## Supplementary Information

Below is the link to the electronic supplementary material.

ESM 1 (DOCX 485 KB)

## Data Availability

No datasets were generated or analysed during the current study.
