# Supplementary material for: Anthropometrics, cancer risks, and survival outcomes in adult patients with glioma – a systematic review and meta-analysis
Source: Acta Neurochir (Wien). 2025 Jul 10;167(1):188. doi: 10.1007/s00701-025-06579-4 (PMC12241189; doi:10.1007/s00701-025-06579-4)
Supplement: Supplementary file 1 — (DOCX 485 KB) [file 701_2025_6579_MOESM1_ESM.docx]

**Supplementary Materials**

Supplementary Figure S1. PRISMA Flow Diagram

Supplementary Figure S2. Sensitivity analysis of the impact of BMI on survival outcomes in glioblastoma and high-grade glioma patients

Supplementary Table S1. List of Studies Excluded During Full Text Assessment

Supplementary Table S2. Findings from Included Studies on Height and Risk of Occurrence in Glioma, Glioblastoma Patients

Supplementary Table S3. Findings From Included Studies on BMI and Risk of Occurrence in Glioma, Glioblastoma Patients

Supplementary Table S4. Findings From Included Studies on BMI and Overall Survival in Glioblastoma and High-Grade Glioma Patients

Supplementary Table S5. Summary of Excluded Studies on BMI and Overall Survival in Glioblastoma Patients

**
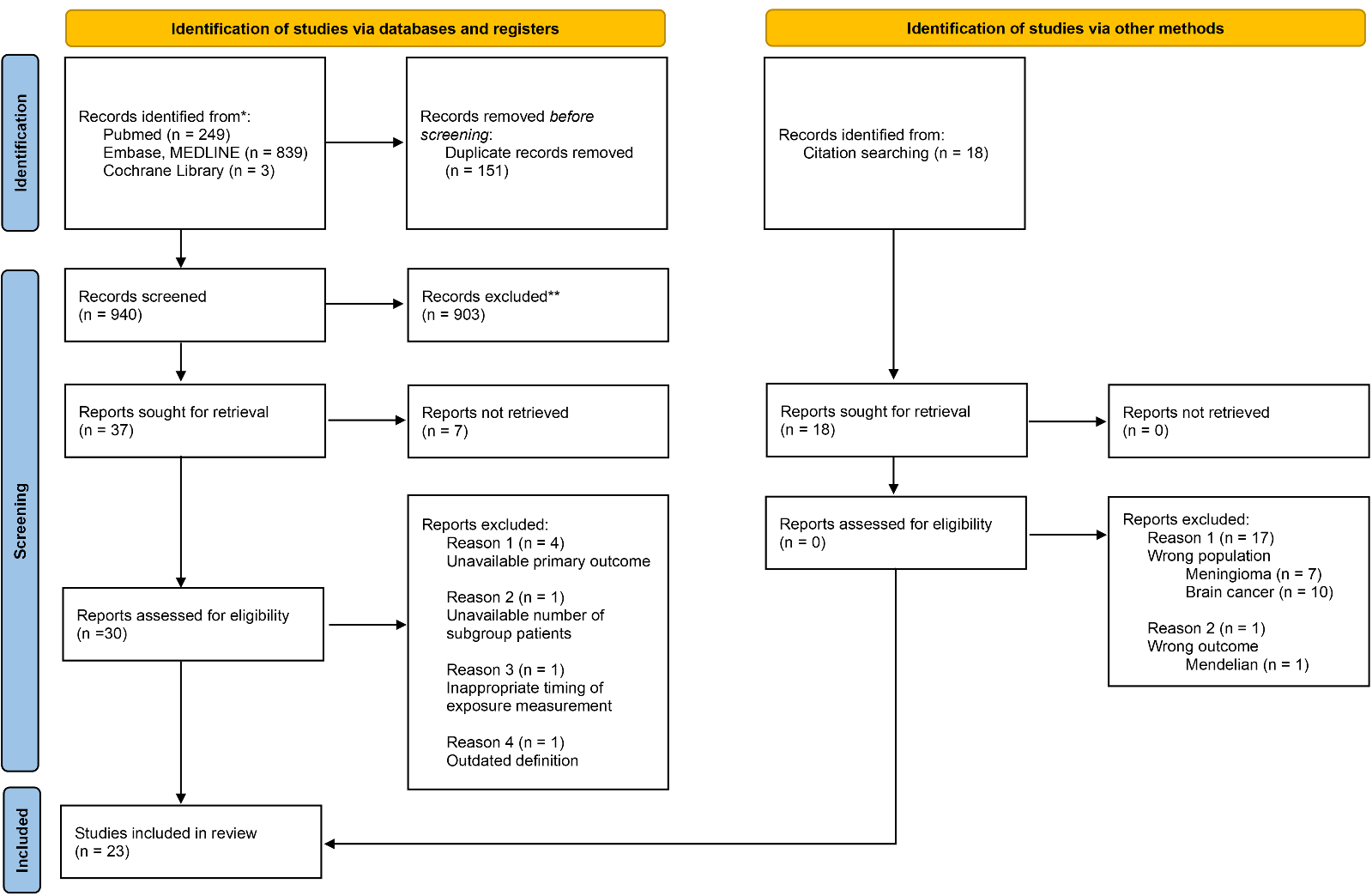
**

**Supplementary Figure S1. PRISMA Flow Diagram**

**
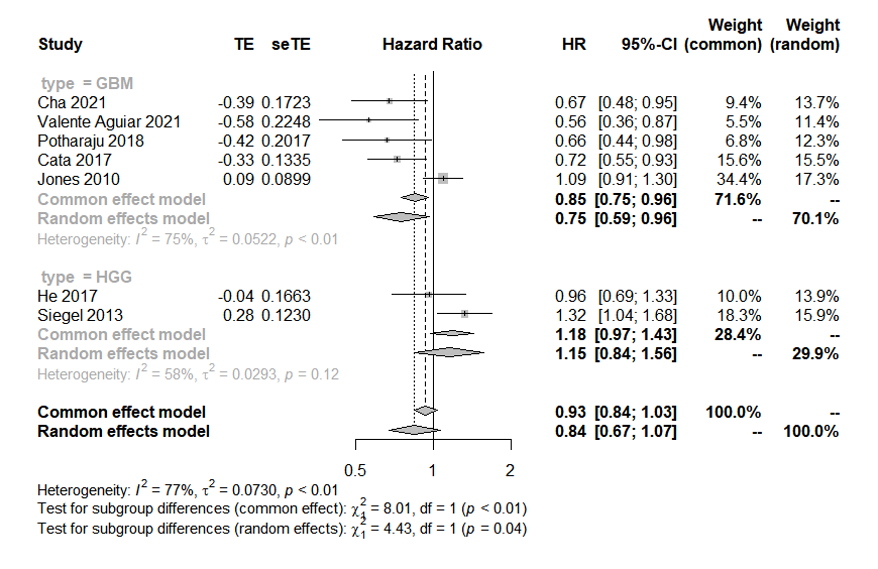
**

**Supplementary Figure S2. Sensitivity analysis of the impact of BMI on survival outcomes in glioblastoma and high-grade glioma patients**

**Supplementary Table S1. List of Studies Excluded During Full Text Assessment**

| Authors | Year | Reason for exclusion |
| --- | --- | --- |
| Weller et al. (1) | 2022 | Hazard ratio unavailable |
| Schneider et al. (2) | 2020 | Hazard ratio unavailable |
| Kitahara et al. (3) | 2014 | Adult anthropometric measures unavailable |
| Chambless et al. (4) | 2012 | Hazard ratio unavailable |
| Edlinger et al. (5) | 2012 | Number of patients by subgroup unavailable |
| Hemminki et al. (6) | 2011 | Hazard ratio unavailable |
| Helseth et al. (7) | 1989 | Outdated definition of brain tumor |

**Supplementary Table S2. Findings from Included Studies on Height and Risk of Occurrence in Glioma, Glioblastoma Patients**

| Authors | Year | Cases (n) | | HR ^a^ (95% CI) per 10 cm | |  |
| --- | --- | --- | --- | --- | --- | --- |
|  |  | Glioma | Glioblastoma | Glioma | Glioblastoma | |
| Ahn et al. (8) ^b^ | 2021 | 4,471 |  | 1.16 (1.1 to 1.22) |  | |
| Cote et al. (9) ^b^ | 2018 | 508 | 321 | 1.31 (1.12 to 1.51) | 1.31 (1.12 to 1.56) | |
| Wiedmann et al. (10, 11) | 2017 | 4,382 | 3,102 | 1.23 (1.17 to 1.29) | 1.24 (1.17 to 1.31) | |
| Little et al. (12) ^b, c^ | 2013 | 1,111 |  | 1.06 (0.93 to 1.21) |  | |
| Wiedmann et al. (13) | 2013 | 148 |  | 1.13 (0.88 to 1.45) |  | |
| Michaud et al. (14) | 2011 | 340 |  | 1.04 (0.87 to 1.24) |  | |
| Moore et al. (15) ^c^ | 2009 | 480 |  | 1.21 (1.07 to 1.37) |  | |
| Benson et al. (16) | 2008 | 646 |  | 1.24 (1.09 to 1.40) |  | |

a Relative risk estimated using Cox models were considered equal to hazard ratio.

b Scale of height was adjusted using log-linear model

c HR stratified by subgroup was combined using inverse variance method

d Analysis only included patients who measured height at 13 years of age

CI, confidence interval; HR, hazard ratio;

**Supplementary Table S3. Findings From Included Studies on BMI and Risk of Occurrence in Glioma, Glioblastoma Patients**

| Authors | Year | Number of Glioma Patients / Total (n) | | |  | RR of BMI ≥ 25kg/m^2^ (95% CI) | |  | HR of BMI +5kg/m^2^ (95% CI) | |  |
| --- | --- | --- | --- | --- | --- | --- | --- | --- | --- | --- | --- |
|  |  | BMI < 25 | 25 ≤ BMI < 30 | BMI ≥ 30 |  | Glioma | Glioblastoma |  | Glioma | Glioblastoma | |
| Moseeva et al. (17) | 2024 | 32 / 16,217 | 8 / 3,898 ^a^ |  |  | 1.04 (0.44 to 2.16) |  |  |  |  | |
| Sang et al. (18) | 2023 | 937 / 976,073 | 910 / 916,984 ^a^ |  |  | 1.03 (0.94 to 1.13) |  |  |  |  | |
| Shao et al. (19) | 2022 | 89 / 47,659 | 127 / 59,545 | 53 / 33,066 |  | 1.04 (0.81 to 1.34) |  |  |  |  | |
| Ahn et al. (20) | 2021 | 2807 / 4,578,686 | 1664 / 2,555,058 ^a^ |  |  | 1.20 (1.13 to 1.28) |  |  |  |  | |
| Ogawa et al. (21) | 2020 | 38 / 74,963 | 22 / 27,962 ^a^ |  |  | 1.55 (0.92 to 2.62) |  |  |  |  | |
| Bertoli et al. (22) | 2018 | 22 / 45 | 21 / 42 | 7 / 15 |  | 1.04 (0.70 to 1.54) |  |  |  |  | |
| Cote et al. (9) | 2018 | 305 / NA | 150 / NA | 49 / NA |  |  |  |  | 1.02 (1.00 to 1.04) | 1.03 (1.00 to 1.06) | |
| Kabat et al. (23) | 2018 | 70 / 54,950 ^b^ | 74 / 55,699 | 68 / 48,387 |  | 1.07 (0.80 to 1.43) | 1.07 (0.77 to 1.48) |  |  |  | |
| Wiedmann et al. (10, 11) | 2017 | 2829 / NA | 1292 / NA | 261 / NA |  |  |  |  | 0.99 (0.94 to 1.03) | 1.00 (0.95 to 1.06) | |
| Little et al. (12) | 2013 | 818 / 1,687 | 221 / 393 | 52 / 101 |  | 1.14 (1.04 to 1.25) |  |  | 1.24 (1.10 to 1.40) |  | |
| Wiedmann et al. (13) | 2013 | 85 / NA | 49 / NA | 14 / NA |  |  |  |  | 1.00 (0.81 to 1.28) |  | |
| Michaud et al. (14) | 2011 | 138 / 174,100 | 147 / 146,300 | 52 / 60,108 |  | 1.23 (0.99 to 1.53) |  |  | 1.02 (0.89 to 1.17) |  | |
| Benson et al. (16) | 2008 | 259 / 548,846 | 241 / 422,508 | 106 / 212,871 |  | 1.16 (0.99 to 1.36) |  |  |  |  | |

a all patients whose BMI ≥ 25 were included in the group

b Only patients with BMI ≥ 18.5 were included

BMI, body mass index; CI, confidence interval; HR, hazard ratio; NA, not available; RR, risk ratio;

**Supplementary Table S4. Summary of Excluded Studies on BMI and Overall Survival in Glioblastoma Patients**

| Authors | Year | Enrollment (n) | |  | Median OS (months, 95% CI) | |  | BMI criteria (kg/m^2^) | |
| --- | --- | --- | --- | --- | --- | --- | --- | --- | --- |
|  |  | Lower BMI | Higher BMI |  | Lower BMI | Higher BMI |  | Lower BMI | Higher BMI |
| Weller et al. (1) | 2022 | 130 ^a^  100 ^b^ | 38 ^a^  29 ^b^ |  | 17.6 (14.7 to 20.8) ^a^  43.2 (32.5 to 54.4) ^b^ | 17.1 (15.8 to 18.9) ^a^  22.9 (17.7 to 30.8) ^b^ |  | < 30 | ≥ 30 |
| Schneider et al. (2) | 2020 | 39 | 28 |  | 10 (7.4 to 12.6) | 15 (12.9 to 17.1) |  | < 25 | ≥ 30 |

a MGMT unmethylated cohort

b MGMT methylated cohort

BMI, body mass index; CI, confidence interval; OS, overall survival;

**Supplementary Table S5. Findings From Included Studies on BMI and Overall Survival in Glioblastoma and High-Grade Glioma Patients**

| Authors | Year | Enrollment (n) | | Hazard Ratio (95% CI) ^a^ | BMI criteria (kg/m^2^) | |
| --- | --- | --- | --- | --- | --- | --- |
|  |  | Lower BMI | Higher BMI |  | Lower BMI | Higher BMI |
| **Glioblastoma** |  |  |  |  |  |  |
| Cha et al. (24) | 2021 | 62 | 115 | 0.67 (0.48 to 0.95) | < 23 | ≥ 23 |
| Valente Aguiar et al. (25) | 2021 | 161 | 32 | 0.56 (0.36 to 0.87) ^b^ | 18.5–29.9 | ≥ 30 |
| Potharaju et al. (26) | 2018 | 209 | 40 | 0.66 (0.45 to 0.98) | 18.5–24.9 | ≥ 30 |
| Cata et al. (27) | 2017 | 106 | 272 | 0.72 (0.55 to 0.93) | ≤ 25 | > 25 |
| Jones et al. (28) | 2010 | 39 | 20 | 1.09 (0.91 to 1.30) | 18.5–24.9 | ≥ 30 |
| **High-grade glioma** |  |  |  |  |  |  |
| He et al. (29) | 2017 | 94 | 94 | 0.96 (0.69 to 1.33) | < 22.2 | ≥ 22.2 |
| Siegel et al. (30) | 2013 | 251 | 241 | 1.32 (1.04 to 1.68) | 18.5–24.9 | ≥ 30 |

a lower BMI group was set to reference

b CI was calculated from p-value

BMI, body mass index; CI, confidence interval;

**References**

1. Weller J, Schäfer N, Schaub C, Potthoff AL, Steinbach JP, Schlegel U, et al. Prognostic impact of obesity in newly-diagnosed glioblastoma: a secondary analysis of CeTeG/NOA-09 and GLARIUS. Journal of Neuro-Oncology. 2022;159(1):95-101.

2. Schneider M, Potthoff AL, Scharnböck E, Heimann M, Schäfer N, Weller J, et al. Newly diagnosed glioblastoma in geriatric (65 +) patients: impact of patients frailty, comorbidity burden and obesity on overall survival. Journal of Neuro-Oncology. 2020;149(3):421-7.

3. Kitahara CM, Gamborg M, Rajaraman P, Sørensen TIA, Baker JL. A prospective study of height and body mass index in childhood, birth weight, and risk of adult glioma over 40 years of follow-up. American Journal of Epidemiology. 2014;180(8):821-9.

4. Chambless LB, Parker SL, Hassam-Malani L, McGirt MJ, Thompson RC. Type 2 diabetes mellitus and obesity are independent risk factors for poor outcome in patients with high-grade glioma. Journal of Neuro-Oncology. 2012;106(2):383-9.

5. Edlinger M, Strohmaier S, Jonsson H, Bjørge T, Manjer J, Borena WT, et al. Blood pressure and other metabolic syndrome factors and risk of brain tumour in the large population-based Me-Can cohort study. Journal of Hypertension. 2012;30(2):290-6.

6. Hemminki K, Li X, Sundquist J, Sundquist K. Obesity and familial obesity and risk of cancer. European Journal of Cancer Prevention. 2011;20(5):438-43.

7. Helseth A, Tretli S. Pre-morbid height and weight as risk factors for development of central nervous system neoplasms. Neuroepidemiology. 1989;8(6):277-82.

8. Ahn S, Han K, Lee JE, Jeun SS, Park YM, Joo W, Yang SH. Association between height and the risk of primary brain malignancy in adults: A nationwide population-based cohort study. Neuro-Oncology Advances. 2021;3(1).

9. Cote DJ, Downer MK, Smith TR, Smith-Warner SA, Egan KM, Stampfer MJ. Height, waist circumference, body mass index, and body somatotype across the life course and risk of glioma. Cancer Causes and Control. 2018;29(8):707-19.

10. Wiedmann MKH, Brunborg C, Di Ieva A, Lindemann K, Johannesen TB, Vatten L, et al. Overweight, obesity and height as risk factors for meningioma, glioma, pituitary adenoma and nerve sheath tumor: a large population-based prospective cohort study. Acta Oncologica. 2017;56(10):1302-9.

11. Wiedmann MKH, Brunborg C, Di Ieva A, Lindemann K, Johannesen TB, Vatten L, et al. The impact of body mass index and height on the risk for glioblastoma and other glioma subgroups: A large prospective cohort study. Neuro-Oncology. 2017;19(7):976-85.

12. Little RB, Madden MH, Thompson RC, Olson JJ, LaRocca RV, Pan E, et al. Anthropometric factors in relation to risk of glioma. Cancer Causes and Control. 2013;24(5):1025-31.

13. Wiedmann M, Brunborg C, Lindemann K, Johannesen TB, Vatten L, Helseth E, Zwart JA. Body mass index and the risk of meningioma, glioma and schwannoma in a large prospective cohort study (The HUNT Study). British Journal of Cancer. 2013;109(1):289-94.

14. Michaud DS, Bové G, Gallo V, Schlehofer B, Tjønneland A, Olsen A, et al. Anthropometric measures, physical activity, and risk of glioma and meningioma in a large prospective cohort study. Cancer Prevention Research. 2011;4(9):1385-92.

15. Moore SC, Rajaraman P, Dubrow R, Darefsky AS, Koebnick C, Hollenbeck A, et al. Height, body mass index, and physical activity in relation to glioma risk. Cancer Research. 2009;69(21):8349-55.

16. Benson VS, Pirie K, Green J, Casabonne D, Beral V. Lifestyle factors and primary glioma and meningioma tumours in the Million Women Study cohort. British Journal of Cancer. 2008;99(1):185-90.

17. Moseeva MB, Azizova TV, Bannikova MV. Risk of central nervous system tumour incidence in a cohort of workers chronically exposed to ionising radiation. Radiation and environmental biophysics. 2024.

18. Sang H, Cho YK, Han K, Koh EH. Impact of abdominal obesity on the risk of glioma development in patients with diabetes: A nationwide population-based cohort study in Korea. PLoS ONE. 2023;18(3 March).

19. Shao C, Tang H, Wang X, He J, Wang P, Wu N. Body mass index and glioma risk: A prospective multicenter study. Frontiers in Endocrinology. 2022;13.

20. Ahn S, Han K, Lee JE, Jeun SS, Park YM, Yang SH. Associations of general and abdominal obesity with the risk of glioma development. Cancers. 2021;13(12).

21. Ogawa T, Sawada N, Iwasaki M, Budhathoki S, Yamaji T, Shimazu T, et al. Body mass index and height in relation to brain tumor risk in a Japanese population. Annals of Epidemiology. 2020;51:1-6.

22. Bertoli S, Battezzati A, Petruzzi A, Leone A, De Amicis R, Tramacere E, et al. Anthropometrics and Body Composition in Adults with High-Grade Gliomas: Effects of Disease-Related Variables. Nutrition and Cancer. 2018;70(3):431-40.

23. Kabat GC, Rohan TE. Adiposity at different periods of life and risk of adult glioma in a cohort of postmenopausal women. Cancer Epidemiology. 2018;54:71-4.

24. Cha JY, Park JS, Hong YK, Jeun SS, Ahn S. Impact of Body Mass Index on Survival Outcome in Patients with Newly Diagnosed Glioblastoma: A Retrospective Single-Center Study. Integrative Cancer Therapies. 2021;20.

25. Valente Aguiar P, Carvalho B, Vaz R, Linhares P. Body mass index as an independent prognostic factor in glioblastoma. Cancer Causes and Control. 2021;32(4):327-36.

26. Potharaju M, Mangaleswaran B, Mathavan A, John R, Thamburaj V, Ghosh S, et al. Body Mass Index as a Prognostic Marker in Glioblastoma Multiforme: A Clinical Outcome. International Journal of Radiation Oncology Biology Physics. 2018;102(1):204-9.

27. Cata JP, Hagan KB, Bhavsar SDO, Arunkumar R, Grasu R, Dang A, et al. The use of isoflurane and desflurane as inhalational agents for glioblastoma surgery. A survival analysis. Journal of Clinical Neuroscience. 2017;35:82-7.

28. Jones LW, Ali-Osman F, Lipp E, Marcello JE, McCarthy B, McCoy L, et al. Association between body mass index and mortality in patients with glioblastoma mutliforme. Cancer Causes and Control. 2010;21(12):2195-201.

29. He ZQ, Ke C, Al-Nahari F, Duan H, Guo CC, Wang Y, et al. Low preoperative prognostic nutritional index predicts poor survival in patients with newly diagnosed high-grade gliomas. J Neurooncol. 2017;132(2):239-47.

30. Siegel EM, Nabors LB, Thompson RC, Olson JJ, Browning JE, Madden MH, et al. Prediagnostic body weight and survival in high grade glioma. Journal of Neuro-Oncology. 2013;114(1):79-84.
